# High resolution ultra-fast sparse sampling with iterative reconstruction imaging for left ventricular evaluation: clinical comparison with standard SSFP imaging

**DOI:** 10.1186/1532-429X-17-S1-O64

**Published:** 2015-02-03

**Authors:** Christian R Hamilton-Craig, Robyn Riley, Andrew Trotter, Johanne Neill, Michael O Zenge, Michaela Schmidt, Benjamin Schmitt, Wendy E Strugnell

**Affiliations:** 1 Centre for Advanced Imaging, University of Queensland, Brisbane, QLD, Australia; 2 Richard Slaughter Centre of Excellence in CVMRI, The Prince Charles Hospital, Brisbane, QLD, Australia; 3 Imaging & Therapy Systems Division, Siemens Healthcare Australia, Brisbane, QLD, Australia; 4 Magnetic Resonance, Siemens Healthcare AG, Erlangen, Germany

## Background

Cardiovascular MRI (CMR) has the advantages of high temporal and spatial resolution with excellent endocardial definition, but ECG-gated cine SSFP imaging requires multiple sequential breath-holds and is time-consuming. Sparse sampling with iterative reconstructionallows for ultra-fast acquisitions of SSFP cine images. The accuracy of this technique compared to standard SSFP imaging requires further validation in the clinical setting.

## Methods

Prospective, observational study of clinical patients referred for CMR imaging for left ventricular evaluation, performed on a Siemens Magnetom Aera 1.5T scanner in a large, tertiary clinical service. All patients had a standard left ventricular short axis stack using standard ECG-triggered TrueFISP imaging (TE 1.3ms, TR 36ms, inplane spatial resolution 1.2 x 1.2 mm^2^), requiring 10-14 breath-holds of 10-15s, and then repeated with an ECG-triggered prototype TrueFISP sequence (TE 1.2ms, TR 31ms, inplane spatial resolution 1.5x1.5 mm^2^) with a constrained undersampling resulting in a net acceleration factor of 4 (CV_sparse), acquired in a single breath-hold or two breath-holds (depending on cardiac size). For the CV_sparse approach, coil sensitivity maps were calculated from the temporal average of the input data in a central region of k-space and image data were then reconstructed using a non-linear iterative reconstruction with k-t regularization of the SENSE type dataset. Datasets were analysed by two experienced readers (>10 years CMR analysis), with papillary muscles and trabeculae included in the blood pool. Comparison of TrueFISP and CV_sparse was assessed using paired *t*-test and Pearson's correlation, and inter-observery reproducibility by Bland-Altman

## Results

**Figure 1:**
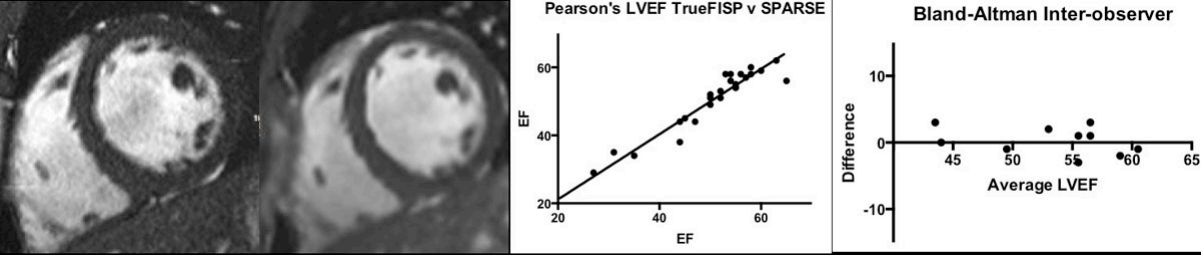
A. TrueFisp B. CV_SPARSE C. Pearson's Correlation; D. Bland-Altman LVEF%

26 clinical patients had both TrueFISP and CV_sparse imaging successfully performed (*figure*1A,B), mean LVEF 49% (range 16-65%). Strong positive correlation was present for end-diastolic volumes (*r*=0.98, P< 0.0001), end-systolic volume (*r*=0.99, P< 0.0001), LVmass (*r*=0.97, P< 0.0001) and LVEF (*r*=0.97, P< 0.0001), with no difference between the groups (*P*=0.95 NS), *figure*1C. Reproducibility was excellent with bias 0.3%, limits-of-agreement -3.7 to 4.3% (*figure*1D). Acquisition time for the LVSAX series was significantly shorter for CV_sparse compared to TrueFISP (*p*<0.001), however CV_sparse images require longer reconstruction times (~5 minutes), during which clinical image acquisition can continue.

## Conclusions

Highly accelerated imaging using TrueFISP with sparse sampling and iterative reconstruction provides excellent image quality with accurate, reproducible results equalling those of standard TrueFISP imaging, but with significantly more rapid acquisition times. This accelerated technique appears valid and ready for application to clinical practice.

## Funding

Smart Futures Fellowship Early Career Grant (Queensland State Government #ISF783).

